# Orchestrating the Development of a Sustainable Network IT Solution for a Research Network: Qualitative Participatory Multimethod Design

**DOI:** 10.2196/82952

**Published:** 2026-04-02

**Authors:** Gülay Ates, Christian Thies, Johanna Schweizer, Arezoo Bozorgmehr, Steve Piller

**Affiliations:** 1 Institute for Digitalization and General Medicine, Center for Rare Diseases Aachen (ZSEA), Medical Faculty, RWTH Aachen University, Pauwelsstraße 30, Aachen, 52074, Germany, 49 0241888 07 1; 2 Reutlingen Research Institute, Reutlingen University, Reutlingen, Germany; 3 Institute of General Medicine, Faculty of Medicine, University of Münster, Münster, Germany; 4 Department of General Practice, Faculty of Medicine and University Hospital Carl Gustav Carus, TUD Dresden University of Technology, Dresden, Germany

**Keywords:** practice-based research networks, PBRN, health information technology, health informatics, screening, primary health care, stakeholder engagement, interoperability, data sharing, health services research

## Abstract

**Background:**

Practice-based research networks (PBRNs) rely on sustainable and interoperable IT infrastructures to support coordination, data management, and long-term collaboration across geographically distributed primary care practices. Large federated initiatives, such as the German DESAM-ForNet (Initiative of German Practice-Based Research Networks) program, face substantial sociotechnical challenges, as diverse user groups, heterogeneous local systems, and multiple governance levels must align around shared digital solutions.

**Objective:**

The aim of this study was to design and evaluate a participatory, consensus-driven process for developing a sustainable and interoperable IT solution that supports the coordination of multiple regional PBRNs, and to identify the sociotechnical factors that influence how such a process unfolds.

**Methods:**

A qualitative participatory multimethod design combined an iterative consensus-based IT development process in a central working group, interdisciplinary domain-driven design workshops (N=40 stakeholders from 6 PBRNs), and qualitative content analysis of internal documents (2020‐2025). Members of the IT working group were nominated by networks based on IT responsibility and strategic involvement; workshop participants represented general practitioners, study nurses, researchers, and coordinators. Documents (meeting minutes, workshop artifacts, and decision logs) were coded inductively by 2 authors to trace sociotechnical dynamics and decision trajectories.

**Results:**

The analysis revealed pronounced differences in IT ambitions, resources, and established practices across the 6 PBRNs (ranging from 2 to 90 person-months), which resulted in divergent expectations and uneven readiness for joint development. This heterogeneity—spanning objectives from simple REDCap (Research Electronic Data Capture; Vanderbilt University) databases to comprehensive digitization strategies—necessitated network-specific bounded contexts within a federated architecture. Through iterative development, stakeholders reached consensus on 6 core use cases (base data management, screening or recruitment processes, study or event participation tracking, management of event participation, accreditation procedures, and standardized communication or data exchange) and 2 national proofs-of-concept: quarterly key performance indicator reporting and pseudonymized practice queries based on a shared core dataset. This collaborative process culminated in a 3-tier practice relationship management infrastructure that integrates local autonomy with central metadata management and connectors to the Medical Informatics Initiative and REDCap, and was endorsed by the steering committee as a scalable compromise balancing interoperability and data sovereignty.

**Conclusions:**

The study shows that developing a national, interoperable IT infrastructure for PBRNs depends as much on social and organizational alignment as it does on technical solutions. Iterative participatory collaboration, transparent governance, and early stakeholder engagement were essential for building shared understanding and trust. Strengthening these relational and organizational elements will be crucial for sustaining future implementation efforts and fully realizing the potential of federated data infrastructures in primary care research.

## Introduction

Practice-based research networks (PBRNs) are collaborative enterprises that bring together primary care practitioners and researchers to address questions directly arising from real-world clinical practice [1]. Originating in the late 19th century, PBRNs have evolved into structured research entities that facilitate systematic data collection, conduct observational studies, and support innovation within general practice [2-6]. International experience has demonstrated the effectiveness of PBRNs in generating evidence-based knowledge, improving care quality, and engaging diverse populations [1,7].

A critical enabler of these networks is a robust IT infrastructure (ITI) [1,7], which supports data integration, communication, and coordination across practices and institutions [8]. While electronic health records (EHRs) in general practices serve primarily clinical functions, the ITI of a PBRN provides the broader technical framework required for research collaboration. This includes data management, harmonization, secure exchange, and support for network-wide activities, such as study coordination or practice engagement [9,10].

Studies in clinical settings have shown that the usability of EHR systems is strongly linked to outcomes such as professional satisfaction, workflow efficiency, and data quality [11,12]. While there is extensive literature on EHR usability in hospital and clinical care settings [13-17], much less is known about the design, implementation, and usability of ITIs that support research networks like PBRNs. Emerging evidence suggests that PBRN ITIs require complex local adaptations [18], that usability perceptions vary among different stakeholder roles [19,20], and that participatory approaches are essential to ensure broad acceptance [20]. However, engaging individuals through these participatory and equitable processes may result in variations in both the time required and the outcomes achieved in reaching organizational goals [21].

**Figure 1.**
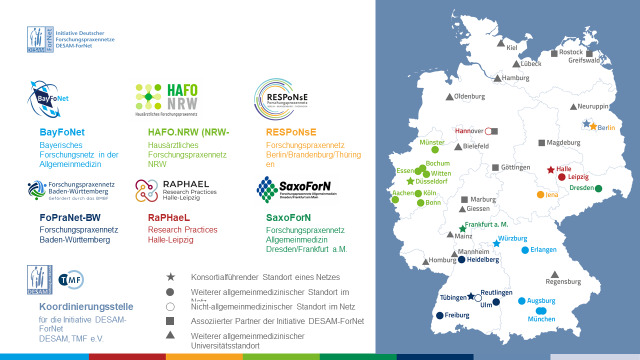
University coordination sites of the general practitioner practices research network.

In Germany, the nationwide DESAM-ForNet initiative [22] represents a major effort to establish PBRNs across all federal states. Between 2020 and 2025, the Federal Ministry of Education and Research funded 6 independent networks—BayForNet, FoPraNet-BW, HAFO.NRW, RaPHaeL, RESPoNsE, and SaxoForN—comprising 27 university departments of general practice and primary care [23]. These networks are forging long-term partnerships with outpatient care providers and are supported by 11 additional, nonfunded associated partners, expanding the initiative’s national reach (Figure 1).

To promote coordination and integration at the national level, the DESAM-ForNet initiative established a central National Coordination Office (NCO) in 2020. This office plays a key role in enabling collaboration across networks, aligning strategic goals, and fostering knowledge exchange at administrative, operational, and scientific levels. A steering group with representatives from all networks ensures participatory governance, balancing institutional autonomy with shared decision-making. Within this governance framework, specialized working groups, such as the IT working group (WG IT), support targeted collaboration on essential components of the initiative.

Although each PBRN has developed independently with its own partners, standards, and pilot studies, the networks have increasingly converged around a shared objective: to create a sustainable, interoperable research infrastructure for general practice. This includes both human and technical components, training programs to prepare general practitioners for research engagement, and digital systems that enable secure data sharing and efficient coordination. A central element of this development is the creation of a practice relationship management (PRM) system, conceptually inspired by customer relationship management (CRM) models, which aims to structure and support long-term partnerships between practices and academic institutions [23].

Developing this infrastructure presents not only technical but also organizational and social challenges [24]. A federated IT system must accommodate a wide range of user needs, from IT specialists and researchers to physicians and administrative staff, each with distinct workflows, expectations, and levels of digital maturity. Prior research on the implementation of health IT systems has consistently shown that sociotechnical factors, such as role ambiguity, communication breakdowns, and insufficient user involvement, can significantly hinder progress [7,25-28]. Governance gaps, unclear strategic direction, and a lack of coordination mechanisms are further risks that may lead to fragmentation, inefficiency, and ultimately, low user acceptance.

Addressing these challenges requires participatory processes [29], early stakeholder engagement, and a shared vision [7,21,25] that aligns technical development with clinical and research priorities. Building mutual trust [30], establishing a common language, and ensuring transparent decision-making are essential steps in navigating the complexities of such a large-scale transformation.

This study, therefore, examines the sociotechnical dynamics underpinning the development of a unified ITI within DESAM-ForNet to identify the factors that facilitate or hinder progress. By doing so, it provides evidence-based insights to improve participatory development processes and enhance stakeholder coordination in large, federated primary care research networks.

## Methods

### Study Design

This study used a qualitative participatory multimethod design combining (1) an iterative consensus-based IT development process, (2) participatory interdisciplinary workshops grounded in domain-driven design (DDD), and (3) a structured qualitative document analysis [7,24,29]. The aim was to collaboratively design and analyze a federated ITI to support the national coordination of 6 regional PBRNs within the DESAM-ForNet initiative in Germany.

### Sampling and Participants

Members of the central WG IT were nominated by each of the 6 PBRNs and the NCO to ensure representation of all relevant perspectives (IT staff, project leads, domain experts, and network coordinators). Selection criteria were (1) formal responsibility for IT or data management in the respective network, (2) involvement in strategic planning of network activities, and (3) willingness to participate in regular virtual meetings over the full project period. Workshop participants were purposively recruited via the network coordinators to cover the main professional groups involved in PBRN work (general practitioners, study nurses, clinicians, IT developers, research staff, and administrative coordinators). Each network was asked to nominate individuals with practical experience in practice-based research and familiarity with existing local IT solutions. This resulted in a sample of 40 multiprofessional stakeholders across all 6 networks.

### Iterative IT Development Process (WG IT)

The WG IT was established in June 2020 to coordinate and harmonize ITI planning across 6 heterogeneous research networks. It consisted of at least 1 IT lead and 1 scientific lead per network plus representatives from the NCO. The group met in structured monthly virtual meetings (60‐90 min) between 2020 and 2025.

The process followed agile principles and comprised repeated cycles of: (1) defining the problem domain and system boundaries, (2) formulating an abstract vision and core requirements, (3) creating and reprioritizing a backlog of tasks and goals, and (4) conducting cycles of analysis, technical design, implementation, and structured review. Each meeting used a standardized agenda (status updates, backlog review, decisions, and next steps) and concluded with explicit documentation of decisions and open issues. Consensus was sought through moderated discussion; if necessary, decisions were postponed to the next meeting after written feedback from all networks.

**Figure 2.**
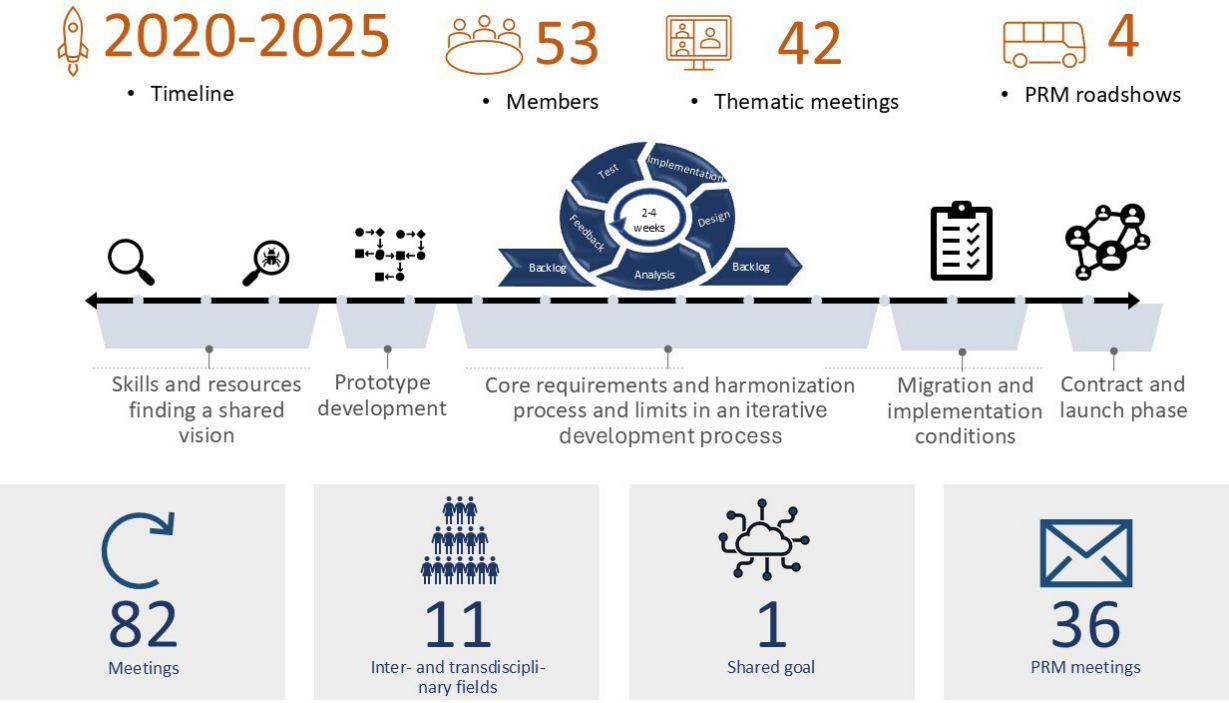
IT working group (WG IT) timeline, process, and sources of data material for analysis. PRM: practice relationship management.

Task and backlog management were conducted within the Nextcloud environment using project management software with version control and change tracking. Minutes, decision logs, and design artifacts were stored in shared, access-restricted folders with date-stamped versions. Figure 2 illustrates the iterative development cycle, the associated meetings, the interdisciplinary engagement, and the core project phases that occurred among the 38 university locations between 2020 and 2025.

### Interdisciplinary Participatory Workshops

Workshops were organized to translate the high-level vision into domain models and concrete use cases. Over a period of 18 months, 6 major workshops (ranging from half-day to full-day) were conducted, alternating between virtual and in-person formats. Each workshop followed a predefined structure: introduction and goal setting, collaborative modeling in mixed-professional groups, plenary consolidation, and agreement on next steps. Two moderators (a project coordinator and an IT facilitator) guided the sessions using structured facilitation methods (eg, brainwriting, small-group work, and dot-voting for prioritization).

The DDD approach was applied to identify bounded contexts, ubiquitous language, and core domains. Participants co-created domain models, user journeys, and use-case descriptions on collaborative whiteboards (eg, Miro) and physical flipcharts during on-site meetings. Consensus on core requirements and use cases was achieved through iterative feedback loops: preliminary models were circulated after each workshop, commented on in writing, and revised in subsequent sessions until no substantial objections remained.

### Data Generation and Documentation

Multiple data sources were generated during the iterative process as follows: (1) minutes and decision protocols from monthly WG IT meetings; (2) workshop agendas, participant lists, and field notes; (3) DDD artifacts, including domain models, user journey maps, and use case descriptions (exported from Miro as PDFs); (4) backlog entries, user stories, and issue tickets from the project management tools and GitLab; and (5) internal resolutions, steering board decisions, grant notices, and network roadshow materials.

All documents were stored in a central Nextcloud instance with role-based access control. Files were named according to a standard convention (date_network_documenttype) and versioned either through the built-in versioning mechanism or explicit version numbers in the file name. For the qualitative analysis, documents from June 2020 to December 2025 were exported, deduplicated, and organized into an analysis corpus by document type and time period.

### Qualitative Document Analysis

Two authors (GA and SP) conducted a qualitative content analysis of the compiled documents. In the first open coding phase, both independently coded a shared subset of documents to identify themes related to design rationale, stakeholder priorities, coordination challenges, and emerging IT concepts. Codes were developed inductively and documented in a shared codebook (Microsoft Excel), which included code labels, definitions, and example passages.

In the second phase, the codebook was refined through discussion and applied to the full corpus by 1 coder, while the second coder reviewed coding summaries and sample documents for consistency. Discrepancies were resolved in consensus meetings, and the codebook was iteratively adapted. In a final phase, codes were grouped into higher-level categories (eg, “heterogeneity of network goals,” “trust and governance,” and “federated architecture decisions”) and mapped against the temporal sequence of meetings and workshops to reconstruct key decision points and development trajectories. No data were missing from the analysis corpus.

### Tools and Technical Environment

Core collaboration and documentation were supported by a central Nextcloud installation (including shared drives, calendar, and task management modules), a project management tool for backlog and issue tracking, GitLab for software development and version control, and Miro for collaborative whiteboarding. For the qualitative content analysis, Microsoft Excel and document management software were used to manage the corpus and codebook. Where relevant, version numbers and configurations of the tools were documented in the project’s internal technical documentation, and all major changes to the tool landscape were recorded in WG IT minutes.

### Ethical Considerations

All internally accessible, anonymized documents from the working group were analyzed with the explicit approval of all WG IT members for research purposes. Networks requested to provide supplementary information were informed about the rationale and scope of the investigation. No formal ethics committee approval was required, as the study involved the analysis of deidentified internal project documents and did not include human participant research as defined by national regulations. All data protection measures adhered to the General Data Protection Regulation and relevant ethical guidelines for online and collaborative data handling, including role-based access controls, pseudonymization of any identifiable content, and secure storage within the project infrastructure.

## Results

### Network-Specific IT Objectives and Bounded Contexts

An initial inventory of IT objectives and available resources across the 6 research networks and the NCO showed marked heterogeneity in ambitions, existing systems, and allocated person-months. Each PBRN pursued its own IT solution and objectives, ranging from comprehensive department-wide platforms to more limited database tools, and had to navigate integration with local systems, established organizational practices, and regulatory requirements (Table 1). These differences motivated the definition of network-specific bounded contexts within an overall federated architecture.

**Table 1.** Overview of IT objectives and resources by network.

Network partner	IT objective	Available resources
NCO^a^	Creating a meta database to collect and provide general information about the regional networks	30 PM^b^ IT, €200,000 (US $231,420) tender
BayFoNet	Using a REDCap^c^ database (Vanderbilt University) to organize practices and studies, providing this information to a website	6 PM IT
HAFO-NRW-GRP	Aiming to develop a seamless data collection instrument (FallAkte Plus) for use in the established German care sector IT infrastructure.	2 PM IT and tender money for support
FoPraNet-BW	Building comprehensive software tools to digitize all processes in the departments and GP^d^ offices (GP practice management or PRM^e^ as well as study planning, monitoring, and execution (CSTM^f^ or PSTM^g^)	90 PM IT
Raphael	Not provided	Not specified
Response	No specific IT goal defined at project start; general support and infrastructure integration	Tender money for support
SaxoForN	Building a CRM^h^-like database to organize network processes (communication, events, and studies)	30 PM IT

a NCO: National Coordination Office.

b PM: person months.

c REDCap: Research Electronic Data Capture.

d GP: general practitioner.

e PRM: practice relationship management.

f CSTM: Clinical Study/Trial Management

g PSTM: Practice Study/Trial Management

h CRM: customer relationship management.

### Harmonization Core Requirements Through the Meta-Database Initiative

The DESAM-ForNet call for tenders for a meta-database created a formal driver to harmonize requirements across networks. The grant specification required compatibility with the Medical Informatics Initiative (MII) core database and adherence to interoperability standards, prompting the WG IT to agree on a common inventory of existing structures before defining a shared core dataset.

Through structured discussions and document reviews, a set of requirements emerged as common to all networks: standardized data management for practices and professionals, support for screening and recruitment processes, accreditation management, and enhanced communication and data exchange. However, the requirements provided by the networks were documented in markedly different formats and levels of abstraction. Some sites submitted structured Microsoft Word or Excel catalogs with functional and nonfunctional requirements or user stories, while others contributed only single high-level needs (eg, a generic interface requirement). In 1 case, requirements were only implicitly represented in an existing PRM prototype. These differences in format, detail, and terminology, together with missing or divergent prioritization schemes, meant that seemingly similar requirements could not be assumed to reflect a shared understanding and required additional clarification. Harmonization was impeded by these heterogeneous documentation practices and varying technical expertise, which led to differing levels of detail and prioritization across networks.

### Common On-Site Use Cases

Qualitative analysis revealed social challenges alongside technical heterogeneity: network leads’ requirements stagnated without operational input, requiring work-level engagement for progress. Despite the heterogeneity, the participatory workshops and document analysis identified 6 core on-site use cases as relevant for all locations: base data management for practices, general practitioners, and study nurses; management of screening and recruitment processes; tracking of study participation; management of event participation; accreditation procedures; and standardized communication and data exchange. The technical implementation of these use cases was explicitly designed to strengthen relationships between sites, standardize processes, and enable cooperation; interfaces to REDCap were included to allow flexible data collection and storage.

Initial requirements submitted by network leads showed limited evolution between their first compilation and the meta-database tender discussions. Substantial progress and alignment only occurred after shifting focus to small-group workshops with work-level staff (researchers, study nurses, and coordinators), where concrete processes were jointly modeled and requirements were refined.

At the national level, 2 cross-network use cases were agreed upon as initial proofs of concept. First, a quarterly key performance indicator (KPI) report summarizing, for example, the number of associated practices and ongoing studies per network. Second, a national practice query based on a consensual core dataset describing structural practice characteristics and qualifications, enabling pseudonymized partner search and characterization of the national PBRN.

### Developing a Harmonized ITI

**Figure 3.**
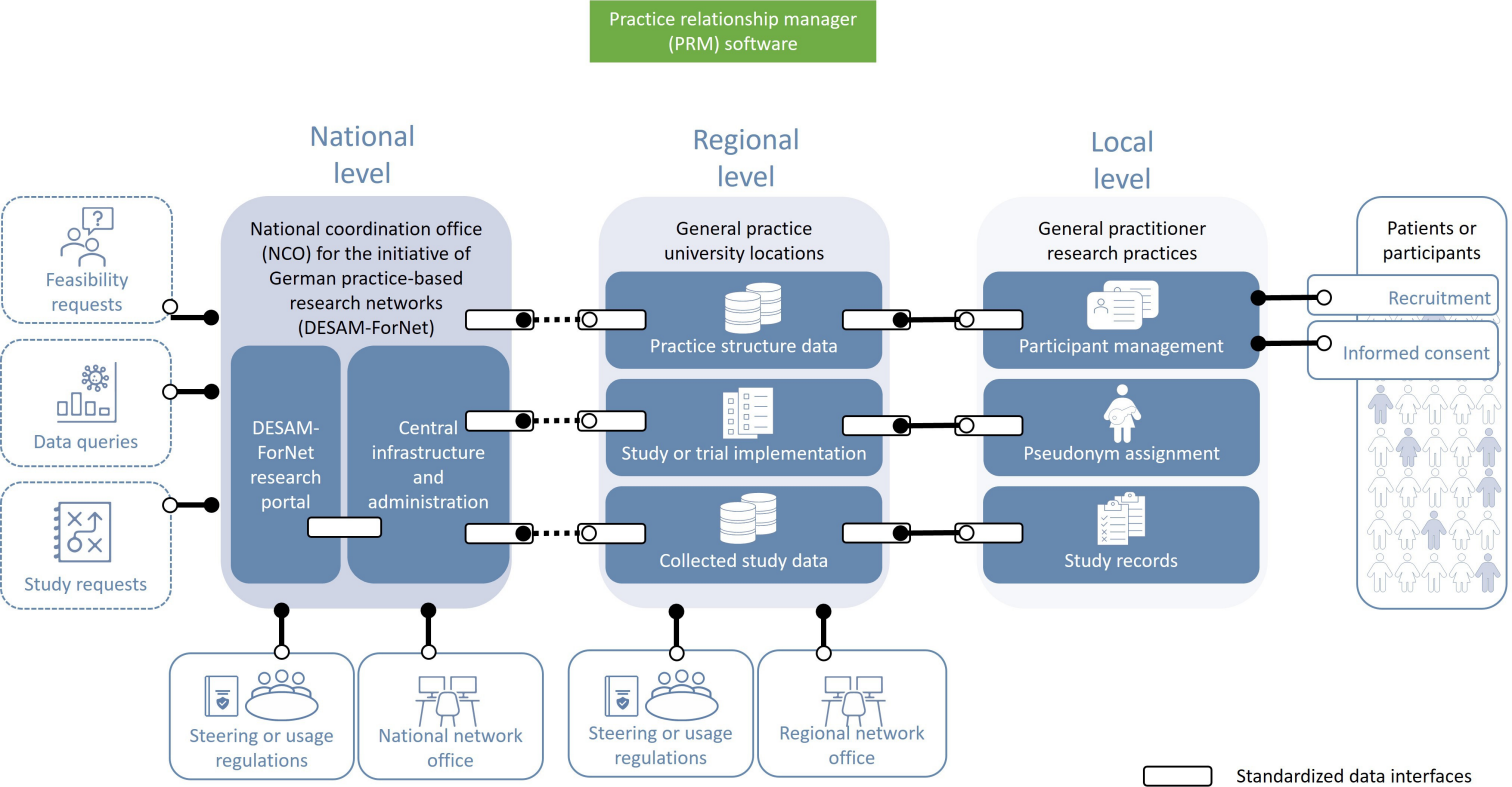
The general 3-tier infrastructure of practice relationship management (PRM). NCO: National Coordination Office.

The process of aligning network-specific objectives and requirements highlighted the need for a common IT foundation, particularly for metadata management. WG IT meeting minutes from October 2020 document the proposal of a 3-tier federal infrastructure model (local network systems → central PRM metadata layer → national MII interfaces), which workshop participants endorsed as a pragmatic solution balancing local flexibility with national coordination needs (Figure 3).

The initial requirements workshop in August 2020 also identified the developmental stages and specific objectives of each research network, resulting in diverse content requirements and IT concepts. Notably, disparities in resources and planned implementations were observed, with some networks compensating for limited resources through increased collaboration.

A significant advantage was the data-oriented nature of the pilot studies, which facilitated the initial harmonization of data structures. Supraregional record linkage was planned as a subsequent step, with pseudonymization and standardized interfaces identified as prerequisites for comprehensive data exchange. Privacy and ethics emerged as critical issues across all networks and were collectively addressed through DESAM-ForNet.

In January 2021, workshops aimed at determining the precise requirements of the general practitioner research network sites concluded that a PRM tool, based on CRM software, would be the most suitable solution. The definition of a common minimum core data set, negotiated in parallel sessions, provided a foundational basis for the meta-database.

From an interoperability perspective, the PRM software was designed with connectors to facilitate future integration of data (Figure 3). For example, the software updates general practice information using data from the German Kassenärztliche Bundesvereinigung, the governing body of the 17 statutory health insurance physician associations in Germany. It can also integrate an external study manager for various study designs and surveys with REDCap servers [10]. Discussions also explored the establishment of a trust center via DESAM-ForNet, although this was not realized within the project timeframe.

A key requirement was that the PRM be user-friendly and accessible to IT laypersons, supporting simultaneous multisite access and intuitive operation. The system was designed to accommodate a generic data-sharing concept, including standardized consent and information documents (broad consent), allowing for resource-efficient updates and adaptations. Data sovereignty remains with each respective network, while the user interface and registration processes are harmonized and integrated into the overall roadmap. The platform enables communication within and between networks, with general practitioners, and with DESAM-ForNet.

### Vision Alignment With the Steering Committee

Based on the iterative consensus-building process, the WG IT submitted a vision to the steering committee that included (1) a simplified version of the FoPraNet-BW infrastructure as a model for national implementation, (2) financing through the tender for the development of a meta-database, and (3) incorporation of national use cases to ensure broad applicability and stakeholder buy-in.

The steering committee endorsed this vision, thereby formally anchoring decentralized data storage combined with centralized infrastructure management as the guiding principle for future development.

### Implementation and Agreement on Core Processes

The initial implementation of the PRM focused on the administration of general practitioner research practices, including the storage of addresses, documentation of recruitment procedures, and tracking of recruitment status. The tool supports the identification of research teams at each general practice, documentation of training and certification, and the management of study invitations and participation.

In 2022, the 6 general practice networks reached an agreement on the storage and documentation of recruitment procedures, the use of 3 core questionnaires for all recruited general practitioners, event management, data exchange for reporting, and documentation of study participation. These standardized processes and questionnaires served as the starting point for the development of the IT solution, ensuring a harmonized and scalable approach across all participating networks. Finally, all implementation steps and agreements on core processes are summarized in Table 2.

**Table 2.** Summary of national use cases and practice relationship management (PRM) functions.

Use case or function	Description or outcome
Key performance indicator report	Quarterly reporting of network and study metrics
National practice query	Searchable, pseudonymized core dataset for GP^a^ recruitment
PRM administration	Management of GP data, recruitment, training, and study tracking
Communication and data exchange	Standardized messaging within or between networks and GPs
Data sharing and consent	Generic, standardized consent processes and broad consent support
Interoperability	Connectors for ePA^b^, MII^c^ integration, and future scalability

a GP: general practitioner.

b ePA: electronic patient record.

c MII: Medical Informatics Initiative.

## Discussion

### Principal Findings

The considerable heterogeneity in IT expertise, occupation, resources, and independently funded strategic objectives among the 6 PBRNs and the NCO posed a significant challenge. This diversity underscores the sociotechnical complexity highlighted in the literature, particularly concerning the development of a nationwide interoperable ITI for primary care research. The findings of this study further confirm these complexities, illustrating the intricate interplay between various stakeholders and their differing capabilities [24-27,31]. Nevertheless, the WG IT succeeded in developing a common vision based on decentralized data storage and centralized infrastructure management. This vision was realized through an IT project that was submitted and financed in the aftermath. The project aimed to facilitate continuous moderate cooperation in the IT area as an ongoing process.

The iterative application of the DDD approach [7] facilitated the identification and harmonization of core requirements unique to a federated, interoperable ITI for primary care research in Germany. This process led to the development of the PRM tool and the definition of national use cases, including KPI reporting and national practice queries. These use cases not only demonstrate the technical feasibility of a federated data infrastructure but also serve as trust-building mechanisms among stakeholders.

The findings align with previous research [24,27,32], highlighting the critical role of social processes—specifically proximity, mediation, and trust—in bridging organizational and cultural differences while fostering receptiveness to new IT solutions. Although the initial lack of a comprehensive IT strategy posed challenges, these were partially addressed through continuous stakeholder engagement, iterative consensus-building, and the establishment of governance structures that effectively balanced local autonomy with national coordination.

The chosen methodology, characterized by intensive communication and regular interaction, facilitated a user-centered development process aligned with Evans’ DDD [7]. The PRM solution effectively maps everyday workflows, offering a user-friendly, open, and interoperable platform tailored to the actual needs of its users. Additionally, the application of CRM-based tools to enhance efficiency in managing PBRN sites is supported by similar improvements observed in other health care domains.

One key challenge was that all grant-funded pilot studies remained network-specific and did not provide practical use cases for the federal infrastructure. Financing the establishment of infrastructure without dedicated pilots to define requirements and demonstrate benefits poses significant risks in federated structures, as the added value may not be immediately evident. Additionally, developing detailed software requirements in the research domain is inherently challenging; researchers often concentrate on high-level requirements, leaving prioritization to developers. Bridging the gap between harmonized requirements and concrete operational specifications necessitated the use of examples and mock-ups, such as the PRM from FoPraNet-BW. Furthermore, trust-building was hindered by the COVID-19 pandemic, which limited opportunities for informal exchanges and relationship development due to the absence of in-person meetings.

Integrating networks with highly variable IT solutions and differing levels of willingness to change remains a significant challenge. Some sites currently use function-oriented software (eg, Microsoft Access) and are hesitant to switch unless clear advantages are demonstrated, while others are eager to adopt new platforms due to limited existing IT support [33]. To fully realize the potential of the IT solution, it is essential to address aspects such as accessibility, intuitive usability, process mapping, and the consideration of hidden user needs.

### Heterogeneity and Sociotechnical Complexity

The documented heterogeneity in IT resources (2‐90 person-months), expertise, and strategic objectives across PBRNs exemplifies the sociotechnical challenges of federated research infrastructures, where local autonomy conflicts with national interoperability needs [24-27,31]. This mirrors experiences in other PBRN networks, where divergent organizational cultures and capacities necessitate bounded contexts to preserve network-specific practices while enabling metadata exchange. The emergence of network-specific IT goals (Table 1) underscores how such disparities can fragment progress unless addressed through structured negotiation.

### Harmonization Through Core Requirements and Use Cases

Despite initial divergences, consensus on 6 core use cases and 2 national proofs-of-concept (KPI reports and practice queries) demonstrates that iterative prioritization can yield shared standards in heterogeneous settings. These agreements align with PBRN good practices emphasizing standardized data management and recruitment tracking as foundational for scalable research. Pronounced structural and organizational differences between networking resources, roles, documentation practices, and local IT solutions—initially slowed progress, as high-level requirements from network leads remained largely unchanged. This barrier was overcome by engaging work-level staff (study assistants and coordinators) in small-group workshops, which developed concrete, shared process understanding and enabled the negotiated core dataset for pseudonymized practice queries. The federated model thus balances data sovereignty with cross-network utility, facilitating targeted collaborations without centralizing sensitive patient data.

### Federated Infrastructure and PRM as Scalable Solutions

The 3-tier PRM architecture—local systems, central metadata layer, MII or REDCap connectors—represents a pragmatic compromise, leveraging existing tools (eg, FoPraNet-BW) for national scalability. This design addresses interoperability gaps identified in German primary care research, where CRM-inspired tools have proven effective for practice coordination. Endorsement by the steering committee validates its feasibility, positioning PRM as a trust-building mechanism that demonstrates immediate value through KPI transparency.

### Methodological Considerations

The DDD approach, applied through interdisciplinary workshops, effectively bridged clinical workflows and technical requirements by establishing a shared ubiquitous language across stakeholders. This user-centered methodology aligns with recent health care IT implementations, where DDD enhances regulatory compliance, reduces role ambiguity, and maps complex domains like recruitment and accreditation. Intensive communication via monthly WG IT meetings further mitigated cultural differences, confirming DDD’s suitability for federated PBRNs despite pandemic-related constraints on in-person trust-building.

### Persistent Challenges and Implications

Network-specific pilot studies highlighted federal use cases, emphasizing the risks of funding infrastructure without embedded research pilots—a pattern observed in other federated networks. Variable IT maturity (eg, Microsoft Access vs comprehensive platforms) and resistance to change remain obstacles that demonstrably require a participatory approach and a focus on user-friendliness. These findings imply that future PBRN IT development should prioritize governance structures, early DDD engagement, and hybrid (virtual or in-person) formats to foster trust and adoption. For DESAM-ForNet and similar initiatives, the PRM model offers a blueprint for integrating with national infrastructures like MII while preserving local flexibility. The DESAM-ForNet process demonstrates strategies applicable beyond Germany, particularly for PBRNs facing similar tensions between local autonomy and national coordination. Lessons for other federated research networks include 3-tier architectures, early engagement of work-level staff to bridge structural heterogeneity, and KPI-driven proofs-of-concept that build trust. These align with international PBRN good practices and could benefit networks coordinated by organizations like the North American Primary Care Research Group. Adaptation remains essential for varying funding models, regulatory frameworks (eg, United States Health Insurance Portability and Accountability Act [HIPAA] vs General Data Protection Regulation), and digital maturity levels, with the PRM model’s modular connectors offering scalability to resource-constrained settings.

### Limitations

#### Exploring New Communication Channels in the Context of the COVID-19 Pandemic

At the beginning of the pandemic, in-person meetings were impossible due to contact restrictions and public health measures. During the early pandemic phase, not all participating institutes initially had access to platforms such as Zoom (Zoom Communications, Inc), which made regular meetings, building trust, and effective collaboration challenging. However, as familiarity with these platforms increased, almost all communication took place online, and the project team quickly adapted to these new formats, ensuring continuity of collaborative work. Consequently, shifts in the timeline and issues of trust emerged in the absence of face-to-face interaction, and these issues manifested themselves in several project phases.

#### Data Processing and Documentation Practices

Although systematic data preparation was facilitated by detailed protocols, these were later streamlined. Furthermore, the heterogeneity of documents across networks posed challenges for comparison, resulting in variability in the depth of documentation between participating networks and necessitating further follow-up and clarification.

#### Participatory Working and Tool Adoption

The project’s participatory nature embraced varying levels of digital proficiency and openness to new tools, such as Miro and Jira. Consequently, some participants continued to submit materials in familiar formats, such as Word documents, Microsoft PowerPoint presentations, and Excel spreadsheets, rather than adopting new collaborative platforms. As a result, the networks differed in how comprehensively they documented activities and outcomes, making the standardization of records challenging and necessitating supplementary inquiries in several cases. Despite these challenges, the project successfully adapted its processes, encouraged collaboration across networks, and established robust protocols to ensure the integrity and reliability of the data collected.

### Conclusions

This study illustrates that the success of large-scale health IT initiatives relies not only on technical factors but also, crucially, on social and organizational processes. Key success factors identified include iterative, participatory development, transparent governance, and the early and continuous engagement of all relevant stakeholders. Additionally, the need for integration with ancillary systems has become increasingly evident and has been proactively addressed in subsequent implementations of transformative systems.

To ensure sustained effectiveness and acceptance of complex health ITIs, it is vital to foster a common vision, establish a shared language, and build mutual trust among all participants [7,28,33]. Consequently, future efforts should prioritize these relational and organizational dimensions alongside technological advancements, aiming to fully leverage the potential of both centralized and federated data management in advancing research and clinical practice.

Large-scale federated ITIs for primary care research networks face inherent sociotechnical tensions between local autonomy and national interoperability. This study demonstrates that participatory, iterative processes can overcome marked heterogeneity in resources, expertise, and objectives across 6 German PBRNs, yielding consensus on core use cases, a shared dataset, and a scalable 3-tier PRM system.

Key success factors include structured governance, continuous stakeholder engagement, and domain-driven co-design, which establish a shared language and trust. These relational mechanisms proved essential for negotiating compromises, such as decentralized data storage with central metadata coordination.

The PRM model offers a blueprint for PBRNs worldwide, balancing flexibility with MII or electronic patient record integration potential. Future initiatives should embed research pilots early, prioritize hybrid trust-building formats, and demonstrate immediate value through KPI transparency to accelerate adoption and sustain long-term collaboration in primary care research.

## Abbreviations

CRM: customer relationship management
DDD: domain-driven design
EHR: electronic health record
HIPAA: Health Insurance Portability and Accountability Act
ITI: IT infrastructure
KPI: key performance indicator
MII: Medical Informatics Initiative
NCO: National Coordination Office
PBRN: practice-based research network
PRM: practice relationship management
REDCap: Research Electronic Data Capture
WG IT: IT working group
